# Cortical allograft strut augmented with platelet-rich plasma for the treatment of long bone non-union in lower limb- a pilot study

**DOI:** 10.1186/s12891-022-05375-w

**Published:** 2022-05-30

**Authors:** Shenghui Wu, Kun Quan, Jiong Mei, Min Dai, Sa Song

**Affiliations:** 1 Department of Orthopaedic Surgery, Shanghai Jiao Tong University Affiliated Sixth People’s Hospital, 600 YiShan Road, Shanghai, 200233 China; 2 Department of Orthopaedic Surgery, The First Affiliated Hospital of Nanchang University, Nanchang, Jiangxi China

**Keywords:** Cortical allograft strut, Platelet-rich plasma, Non-union, Lower limb, Long bone

## Abstract

**Background:**

The autogenous iliac bone graft is the first choice of surgical treatment for long bone non-union. However, many factors limit the use of autogenous bone, such as insufficient bone harvest and complications in the donor site. This study aimed to pilot-test the effectiveness of the cortical allograft strut augmented with Platelet-rich plasma (PRP) on long bone non-union in the lower limb.

**Method:**

This study was a one-armed pilot trial, with thirteen men and four women patients scheduled for surgery. Revision surgery for managing long bone non-union included debridement, internal fixation of the cortical allograft strut, and adding PRP in the fracture site. After surgery, outcome measurements of healing rate, healing time, the incidence of revision, and complications, were assessed at least one-year follow-up.

**Results:**

Fourteen of seventeen participants completed all follow-ups. The mean age of 14 patients was 35.9 years (range, 18–56 years), and the mean BMI was 22.44 ± 1.53 kg/m^2^. All nonunions united after the operation. The mean healing time was 4.6 ± 0.7 months. There was no revision or complication.

**Conclusion:**

Cortical allograft strut augmented with PRP led to healing long bone non-union in the lower limb. More clinical research is required before widespread use.

## Introduction

Bone non-union is a post-surgery complication after a long bone fracture, and the incidence is about 5–10% [1]. Effective treatment of long bone nonunion, especially refractory nonunion, remains a considerable clinical challenge. Of note, the autogenous iliac bone graft is still the gold standard for treating bone non-union [2]. However, not all non-union can be cured at one operation, and even non-union has not been cured with autologous bone grafting for multiple revisions. Inadequate supply of autogenous bone resources precludes clinical transplantation for most bone grafting failure patients. In addition, a considerable proportion of patients refuse harvest iliac bone grafting.

Although autogenous iliac bone harvest is a relatively simple operation, the incidence of complications in the donor site is not rare, 0.76 to 25% [3, 4]. In addition to the limited donor resources, iliac crest fractures and gait disorders may occur in severe cases [5]. Hence, the demands on bone grafting in treating long bone non-union provide the basis for new lines of investigation into the autogenous bone substitutes.

The cortical strut allograft was generally used for advanced age patients, severe osteoporosis, bone defect, short transverse fracture and the discontinuity of medial femoral cortex, especially the position where the plate could not be placed due to proximity to certain vessels and nerves [6]. Cortical allografts were generally larger and longer than autografts in size and, therefore, can be used in more significant defects [7]. Although allogeneic bone is not comparable to the autogenous bone in osteogenesis, bone conductivity, bone induction, and fixation stability, allogeneic bone is accessible [8]. Moreover, studies have shown that cortical allograft struts, whether alone or in combination with a plate or cancellous bone graft, were valuable auxiliary material for treating bone non-union [9].

Platelet-rich plasma (PRP) has been widely used in studies focusing on different potential indications, with encouraging results [10]. Although patients undergoing treatment for knee osteoarthritis with PRP can be expected to experience improved clinical outcomes compared with hyaluronic acid patients, the findings of previous studies do not support the use of PRP for the management of knee osteoarthritis and chronic midportion Achilles tendinopathy [11–13]. However, PRP was a new bone induction therapy used as auxiliary bone healing. The application of platelet-associated growth factors in vitro and in vivo has been proved to promote the proliferation of osteoblasts [14]. Meanwhile, PRP enhanced the repair process of delayed bone healing, which was valid only when used in conjunction with bone substitutes [15].

The present study aims to assess the effects of cortical allograft strut augmented with PRP on long bone non-union in the lower limb. We hypothesized that cortical allograft strut augmented with PRP could promote the healing of long bone non-union in the lower limb.

## Methods

### Study design and participants

This single-arm pilot study included patients over 18 years old with long bone shaft aseptic non-union of the tibia or femur. Potential participants with long bone non-union of the lower limb were recruited from a large level-I trauma center. Nonunion was defined as a fracture that persists for a minimum of 9 months without signs of healing for at least 3 months [16]. The exclusion criteria were: patients with infective non-union, primary open fracture, initial pathological fracture, rheumatism, skeletal hypoplasia. The fracture pattern and nature of the non-union were described as AO/OTA [17] and Weber-Cech [18] classification, respectively. The study was approved by the medical research ethics committee and registered at The National Trial Register (07/26/2019, ChiCTR1900024740). For each participant, informed consent was obtained. All patients had inappropriate walking or weight-bearing pain before surgery. No patient had a history of infection at the fracture site.

### Procedure

After exposing the fracture site, the adhesion between the callus and the adjacent soft tissue was not separated, and the sharp rasp was used to rendezvous from the healthy bone to the broken end along the long axis of the shaft so that the protruding callus and the connected peripheral soft tissue remained attached. Then cleaned the fractured site and removed all soft tissue inside the medullary cavity. Although care was taken to minimize soft tissue stripping from bone callus, some soft tissue stripping must have been the requirement for placement of the bone strut.

After the internal fixation, bone grafts were performed to ensure adequate bone grafting inside and outside the medullary cavity. PRP was prepared using the WEGO Platelet-Rich Plasma product (WEGO Ltd., Shandong, China). 5 ml PRP and 0.5 ml thrombin were injected into small containers containing allogeneic elements freeze-dried cancellous bone in vitro. Then mixed and stirred well. The ten cubic centimeters of cancellous bone (Junkang Ltd., Shanghai, China) mixed PRP was implanted into the fracture site, and a 10 cm deep-frozen cortical allograft strut (Aorui Ltd., Shanxi, China) was placed in the more significant defect and fixed with two cerclage wires.

Range of motion of joint and isometric contraction muscle exercises was started from the first postoperative day. Each patient was allowed to walk with crutches for 2 weeks but no weight-bearing on the involved leg. Partial weight-bearing was allowed after 2 weeks and gradually increased until the radiographic criteria for early consolidation were met. Full weight-bearing was allowed 6–8 weeks later.

### Outcome measurements

The non-union was judged to be healed when painless total weight-bearing without macro mobility at fracture site associated with bridging callus formation across 3 of the 4 cortices on anteroposterior and lateral radiographic views [19, 20]. Three observers were used to assess follow-up radiographs for the bone union and were blinded to patient outcomes. In cases of disagreement between the two raters, a third independent rater evaluated bone union.

### Statistical analysis

Sample size (*n* = 17) was calculated by using power calculation software (G*Power, Heinrich-Heine-University, Düsseldorf), by using a two-tailed alpha of 0.05, a beta of 80%, a drop-out rate of 10%, and an estimated difference of (5.4–5) ±0.5 months in the healing time of long bone non-union in the lower limb. This difference was estimated using an expected clinically relevant healing time from relevant clinical studies [21, 22] combined with the results from a database of the level-I trauma center based on autogenous bone-grafting. Descriptive statistical methods (mean and range or standard deviation, sd) were used to evaluate the data. SPSS statistical software [SPSS Statistics 20.0 (SPSS Inc., Chicago, IL, USA)] was used for data acquisition and analysis.

## Results

**Fig. 1:**
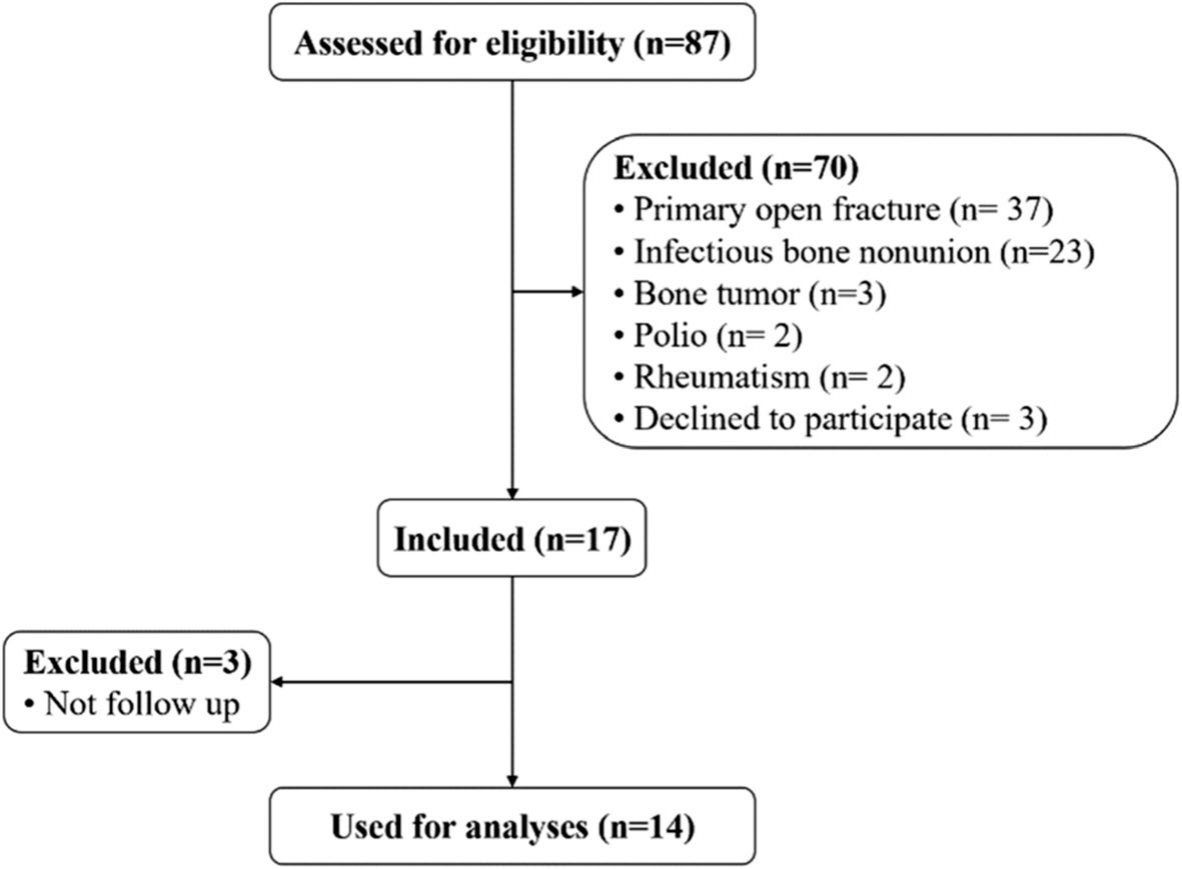
Flowchart of participants

A total of 17 participants were enrolled in this study (Fig. 1). Three participants were excluded due to no follow-up. The remaining 14 participants completed all follow-ups. The mean follow-up was 30.9 months (sd 8.7). Baseline characteristics of the 14 patients were presented in Table 1. The outcome of long bone nonunion treated with cortical allograft strut augmented with PRP was shown in Table 2. All patients healed within 6 months after the operation, and there were no complications such as infection, immune reaction, malunion, or graft fracture. The activity of patients after the operation was better than that before the surgery and walked freely.

**Table 1:** Characteristics and clinical data of 14 patients

Variables	
Age (year)	18–56
Male/Female (No.)	11/3
Site (femur/tibia) (No.)	7/7
BMI (kg/m^2^)	22.44 ± 1.53
Fracture type (No.)	
AO 32 A1/A2/A3	1/1/1
AO 32 B2/B3	1/1
AO 32 C2/C3	0/2
AO 42 A1/A2/A3	0/1/1
AO 42 B2/B3	1/2
AO 42 C2/C3	0/2
Nonunion type (No.)	
Hypertrophic nonunion	3
Hypotrophic nonunion	9
Atrophic nonunion	2
Duration of nonunion (month)	9–37
Past revision frequency 0/1/2/3	7/5/1/1
Total surgery frequency (No.)	38
Final internal fixation change (No.)	10
Exchange intramedullary nail	1
Intramedullary nail plus plate	2
Intramedullary nail to plate	0
Exchange plate	0
Plate to intramedullary nail	6
Plate plus plate	1

**Table 2:** The outcome of long bone nonunion treated with cortical allograft strut augmented with PRP

Variables	
Healing time (month)	4.6 ± 0.7
Healing rate(%)	100%
Nonunion complication	None
Grafting morbidity	None

**Fig. 2:**
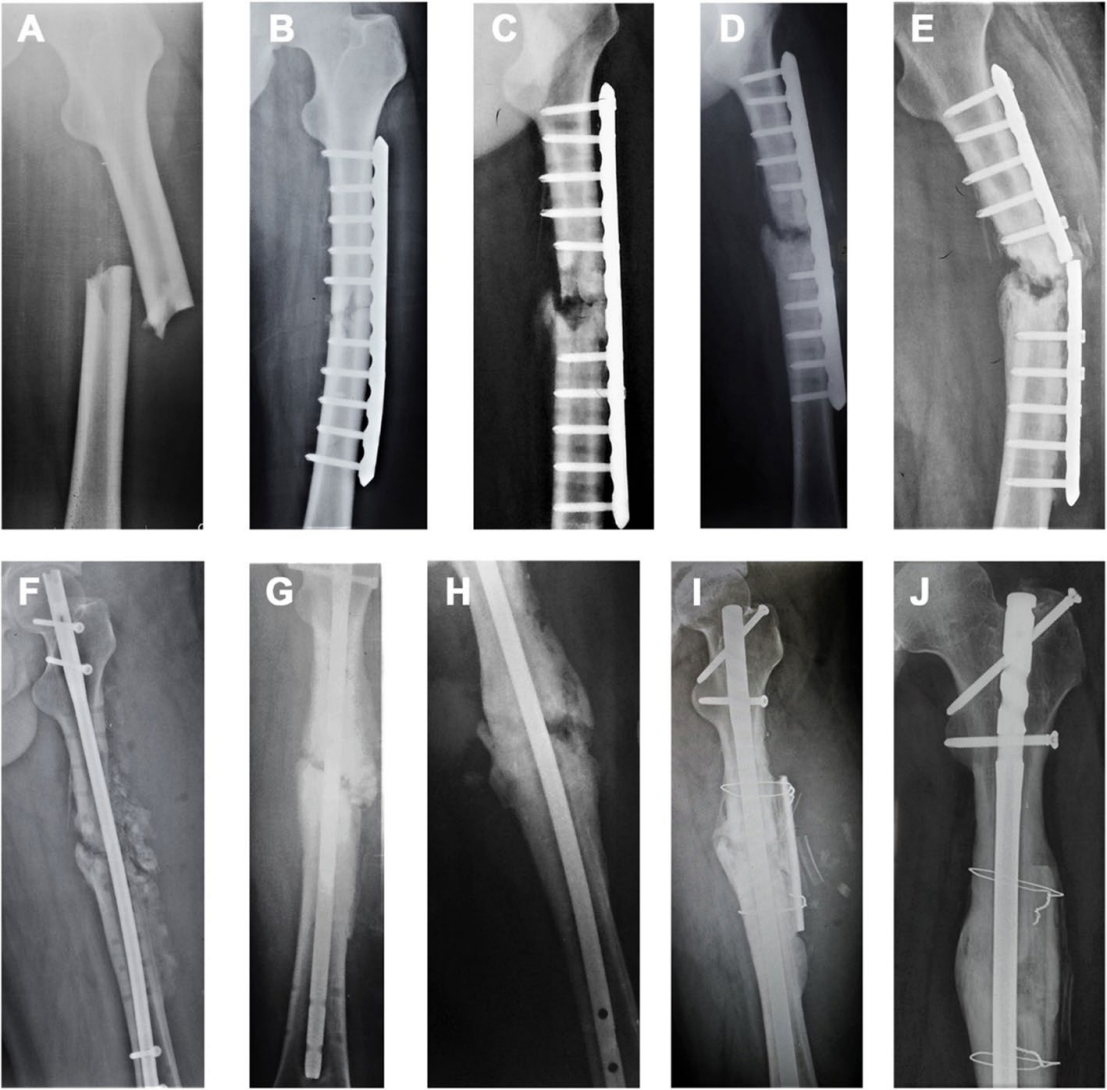
**A** male, 26 years old, non-union of left femur shaft for 23 months. A Displaced fracture of the left femoral shaft caused by a traffic accident. **B** Open reduction and internal fixation postoperatively. **C** Six months after the operation, the plate loosed. **D** The plate was re-fixed and bone grafted with autologous bone. **E** Six months after the second operation, the plate broke. **F** Intramedullary nail fixation and bone grafting with autologous bone. **G** Within 5 months after the third operation, the non-union site did not heal. And intramedullary nails dynamization was conducted with autologous bone grafting. **H** After the fourth operation, the non-union site still did not unite. **I** Intramedullary nail fixation. Due to the limited resources of the donor site, cortical allograft strut grafting was finally performed. **J** Allogeneic bone fusion and nonunion healing in 18 months after the final operation, and the shape was “biconvex.” The contralateral cortical site healed completely, and there was a creeping substitution between cortical allograft strut and femur. The periosteum of the femur has wholly crossed the strut

The healing way of the study group showed a “double convex” shape, with the thickness of the cortex at the side of the strut and the contralateral side thicker than the surrounding cortex. The strut was fused with the autogenous bone but not entirely replaced by the autogenous bone. An epiphysis wholly replaced the site of non-union. (Fig. 2 A-J).

## Discussion

The treatment of long bone nonunion remains a considerable challenge for orthopaedists. This study showed that cortical allograft strut augmented with platelet-rich plasma could successfully treat long bone nonunions in the lower limb. Although autografts were fresh viable tissues with cancellous parts and the bone marrow, which were not only osteoconductive but also osteoinductive and osteogenic grafts, cortical allografts also have some superiority to autografts in terms of larger and longer size and more significant defects [7]. These echoed the findings of earlier research, and PRP seemed, to some extent, an attractive biological to enhance fracture healing [23]. The bone healing of nonunion on the midshaft clavicle, distal femur, forearm, humeral shafts, distal femoral was successfully reported by treating with cortical allograft struts [21, 22, 24–26]. Hence, cortical allograft strut augmented with platelet-rich plasma might be another possible treatment option for long bone shaft nonunion.

The occurrence of long bone non-union in lower limbs may be related to insufficient stability and biological environment disorders. Massive trauma, large fracture gap, unstable internal fixation of the fracture, premature loosening, infection, extensive osteonecrosis, comorbidities, insufficient blood supply, and advanced age are all factors that are not conducive to bone healing [27]. And the management of any non-union involves two critical components of fracture healing: structural support and biology [21]. Autogenous bone contains high-quality osteoconductive, osteoinductive, osteogenic properties and increases the stability of the fracture site, so the efficacy of revision for non-union combined with autologous bone grafting is worthy of recognition. However, limited donor site resources and donor site complications limit the widespread use of autografts. Patients with prior failure of revisions have limited donor site resources and refuse autologous bone harvesting due to possible donor site complications. Hence, to avoid the problems of donor site complications and limited donor site resources, more and more studies are looking for alternative materials or factors with similar properties to treat non-union.

Deep frozen cortical allograft struts retain their material properties and can be implanted immediately after thawing [8]. Moreover,  the allogeneic bone has a certain degree of osteoinduction, bone conduction, and fixation stability. However, the graft exerts biological activity in isolation, depending on the surrounding environment. The cells respond to their signals and also rely on the blood supply. Moreover, the allogeneic bone lacks osteogenic and angiogenic factors. However, despite some studies having achieved excellent results using a small amount of autogenous bone, donor site complications could not be entirely avoided using autografts.

Moreover, platelet-derived growth factors are a class of signaling molecules that participate in the cell repair process by activating the proliferation and chemotaxis of mesenchymal cells, osteoblasts, and chondrocytes [28]. After activation, PRP releases many growth factors, supplementing the allogeneic bone with insufficient activity. This study activated PRP in vitro and implanted it into the non-union site to avoid reducing the local concentration of PRP and thrombin due to the presence of local body fluids and the loss of a large number of released growth factors in the stroma. In addition, the cancellous bone contains many cavities, which can be used as a PRP carrier, and PRP can be used more efficiently.

This study showed that cortical allograft strut augmented with platelet-rich plasma promoted the rapid healing of refractory non-union. This technique may encourage the healing of long bone non-union, regardless of whether it was performed for the first time. Studies have shown that internal fixation combined with allogeneic cortical bone scaffolds alone or with autogenous bone or allogeneic cancellous bone is an active auxiliary material for treating non-union of the femur, humerus and forearm [9, 22, 26, 29]. Besides, previous studies have shown that PRP could promote long bone non-union [30, 31]. Wang et al. [22] treated 13 patients with non-union of the distal femur with allograft cortical bone combined with autologous bone grafting, and the average healing time was 5 months. Kanakeshwar et al. [21] used an allogeneic cortical bone plate combined with autogenous iliac bone grafting and locking plate osteosynthesis in 22 patients with refractory bone non-union at the distal femur. The average healing time was 6.2 months, while the average healing time in this study was 4.6 months. Therefore, this pilot study showed that cortical allograft strut augmented with platelet-rich plasma could improve the healing of long bone non-union of the lower limb. Compared to the previous studies, the healing time in this study was significantly shortened, which may be related to PRP promotion. Additionally, conserving soft tissue and avoiding stripping the bone would have preserved blood supply and aided healing [32].

Different healing ways of long bone nonunion were shown using the cortical allograft strut combined with platelet-rich plasma, and grafting morbidity were not found. This study used a cortical allograft strut with a similar elastic modulus as the host bone. As a “biological plate,” it not only increases mechanical stability but also promotes healing. Meanwhile, cortical allograft strut could be placed in a position that is not suitable for plate placement due to the proximity to certain vessels and nerves. By observing the final healing images of 14 patients, we found that in the way of non-union healing, the shape of the study group was “double convex.” Callus formation on the fracture site’s opposite side of the cortical allograft strut preceded the strut side. There was a creeping replacement process between the cortical allograft strut and the autogenous bone, gradually fused with the autogenous bone. Still, the cortex of allograft bone was not entirely replaced. Besides, if the internal plate fixation were added, the bone cortex in the fracture site at the plate side would heal slowly. Judas et al. [9] used a metal plate combined with allograft cortical bone plate and cancellous bone graft to treat 1 case of non-union of the femur, which healed in 8 months after the operation. Studies have shown that the callus gradually forms at the junction, extending from the periosteum surface of the natural bone to the outer surface of the allogeneic cortical bone. There is a certain degree of creep replacement at the junction of the allograft host, but most of the cortical pillars still die, with an intact structure. On the outer surface of the allograft, the proliferation of mesenchymal cells from adjacent host cells results in a thin layer of bone formation incorporated into the allograft cortex. In addition, grafting morbidity was not found in this study. The previous research has reported the complete resorption of the allografts, and the resorption rate increases proportionally to the follow-up duration in the revision of femoral stems. However, the toughest bone would probably take longer to resorb and provide maximum stability [33].

There are some limitations to this study. First, this was a one-armed pilot trial. Hence, Randomized Controlled Trails and more cohort studies are needed to verify its efficacy. Second, this study did not measure PRP concentrations in individual patients. Although platelet concentrations were not measured in this study, PRP varied according to different people’s blood characteristics and cannot be compared. The PRP used in this study contained a small number of red blood cells. The red blood cell layer shows a small number of platelets. When the plasma and red blood cells are initially separated, retaining a part of the red blood cell layer is beneficial to collect all platelets and reduce platelet loss. Moreover, there are currently more than 16 commercial PRP systems on the market, and there are considerable differences in PRP collection and preparation schemes [15]. The variability in the cellular composition of PRP preparations presents challenges to the literature explaining the clinical efficacy of PRP. Third, the study didn’t use any functional outcome to support this result. However, the most crucial function in the diaphysis of a long bone is maintaining its proximal and distal joints in their correct spatial relationship and providing attachment for muscles that move them. Thus, clinical and radiographic healing plays a vital part in functional outcomes. To the best of our knowledge, there is a lack of consensed clinical function index to assess the prognosis of femoral shaft and tibial shaft fractures.

To conclude, this study shows that it is possible to improve the healing of nonunion in the lower limb by using an allograft strut augmented with platelet-rich plasma. Future studies are needed to confirm the findings of this pilot study versus a control group in patients with long bone nonunion.

## Data Availability

All data generated or analyzed during this study are included in this published article.
